# Using Digital Concept Maps in Conflict Resolution Studies: Implications for Students’ Argumentative Skills, Domain-Specific Knowledge, and Academic Efficacy

**DOI:** 10.3389/fpsyg.2022.882370

**Published:** 2022-07-06

**Authors:** Yoav Kapshuk, Dorit Alt

**Affiliations:** ^1^Kinneret College on the Sea of Galilee, Jordan Valley, Israel; ^2^Tel-Hai College, Qiryat Shemona, Israel

**Keywords:** argumentation for learning, concept mapping, domain-specific knowledge, academic efficacy, higher education

## Abstract

While argumentation emerges as one of the major learning skills in the twenty-first century, a somewhat opaque landscape is revealed in terms of identifying its potential in enhancing higher-education students’ domain-specific knowledge. In this study, argumentation-for-learning activity with digital concept mapping (CM) was designed and compared with a traditional teacher-centered activity to determine the former’s effectiveness in promoting students’ domain-specific factual, conceptual, and procedural knowledge. This study also examines how the proposed activity may contribute to students’ academic efficacy and thus promote meaningful learning. A quasi-experimental design was employed by using convenience samples. Two identical courses were selected for this research: the first course with a total of 59 students (the research group), and the second course including a total of 63 students (the control group). Both groups’ domain-specific knowledge was assessed before and after the activity. The designed activity was found to be less effective in fostering factual knowledge and more effective in developing the conceptual and procedural knowledge domains. Another finding demonstrated the benefits of argumentation for learning with CM in facilitating students’ academic efficacy. It can be concluded that engaging students in a deep argumentation learning process may in turn deepen predominantly conceptual and procedural domain-specific knowledge. Limitations and implications are discussed.

## Introduction

Fostering domain-specific knowledge acquisition is considered a key factor in guiding students toward productive activities and learning (Valero Haro et al., 2019a). This study focuses on students’ domain-specific factual (basic elements related to the discipline), conceptual (understanding of concepts, similarities, and patterns), and procedural (knowing “how” to do something) knowledge (Anderson et al., 2001). Nurturing learners’ domain-specific knowledge is considered valuable, as it might move them beyond basic comprehension to a deeper understanding and enable them to move flexibly between domains (McCarthy and Goldman, 2019); increase their achievements (Chu et al., 2016; Geary et al., 2017); move them toward more scientific and sophisticated reasoning about the discipline (McNeill and Krajcik, 2009); and might enable them to reason about a variety of both familiar and novel problems (Duncan, 2007; von Aufschnaiter et al., 2008). Scaffolding domain-specific knowledge is necessary to help students incorporate evidence into scientific tasks (Ruppert et al., 2019), and might enable them to acquire intellectual skills (Tricot and Sweller, 2013).

These findings have important instructional implications, specifically regarding the need to spur students’ domain-specific knowledge. However, it is not yet clear how to best achieve this learning outcome. Nonetheless, several researchers support the claim that computer-supported argumentation may improve students’ domain-specific knowledge (Asterhan and Schwarz, 2016; Valero Haro et al., 2019a,b, 2022; Ackermann and Kavadarli, 2022). Argumentation, a process of rationally resolving divergent opinions and issues in critical discussions, emerges as one of the major skills for learning in the twenty-first century (Noroozi et al., 2012). For higher education students, learning through computer-supported argumentation is considered essential as it improves the quality of their argumentative writing (Valero Haro et al., 2019a), and their argumentation ability (Fan et al., 2020); enables them to construct knowledge and transfer this knowledge to future situations (Valero Haro et al., 2019a); develops their critical thinking ability (Andrews, 2015; Giri and Paily, 2020), and their ability to actively engage in discussions of socio-scientific issues, a focal learning outcome of higher education studies (Tsai, 2018). Engaging learners in dialogic argumentation, in which they build arguments, consider, and weigh counter arguments, is an important tool to enhance their domain-specific knowledge (Valero Haro et al., 2022).

However, despite increased interest in and writing about learning through argumentation in higher education (Yaman, 2018; von der Mühlen et al., 2019; Chen et al., 2020), prior studies fall short of comprehensively addressing the links between computer-supported argumentation and domain-specific factual, conceptual, and procedural knowledge gains in higher education settings (Weinberger et al., 2017; Kimmerle et al., 2021), and literature in this field that addresses the three domain-specific knowledge facets remains sparse.

To bridge these gaps, in this quasi-experimental research, a technology-enabled concept mapping (CM) method (Novak and Gowin, 1984; Machado and Carvalho, 2020) was used to facilitate argumentation processes, in conflict resolution studies. CM is a visual representation of knowledge. It can be used to organize and structure information within a particular domain. This may be done in a wholly graphical manner to highlight differing concepts and their linkages or by identifying key concepts by names or titles and enclosing them in visual boxes, and then providing connecting navigation to lesser concepts The idea is based on the constructivist approach to learning, which highlights the active role played by learners in constructing and developing knowledge (Alt, 2016).

A few studies (e.g., Chang et al., 2022) showed the benefits of using online CM to facilitate knowledge construction in collaborative learning and improve learning achievement and motivation. Research has also been increasingly devoted to investigating the effectiveness of collaborative CM by which students co-construct their shared knowledge in an active manner (Chi and Wylie, 2014; Lin et al., 2016). Although CM can easily be utilized to co-construct arguments (Medvedeva and Recuber, 2016; Si et al., 2019), literature in the area of argumentation design through CM and the contribution of this learning and instruction method to students’ three domain-specific knowledge facets remains ancillary in higher-education teaching.

In the current study, an argumentation-for-learning activity with digital CM was compared with a traditional teacher-centered activity to determine the former’s effectiveness in promoting students’ domain-specific factual, conceptual, and procedural knowledge (Anderson et al., 2001). The current study also probed how the proposed activity may contribute to students’ academic efficacy, an underexplored variable in this context, considered a key factor that might promote and improve meaningful learning (Bressington et al., 2018). This study offers a techno-pedagogical activity that supports argumentative skills and shows its effectiveness in enhancing students’ domain-specific knowledge and their academic efficacy.

## Literature Review

### Argumentation for Learning

*Argumentation* is considered one of the most valued outcomes of academic programs (Fischer et al., 2018). It has numerous definitions in the literature. Toulmin (2003) defined argumentation as a process in which assertions are produced and justifications are given by way of evidence. Walton (2006) views argumentation as an interactive dialogue in which participants advance arguments by proving or refuting assumptions. Despite the differences, all these definitions point to the use of argumentation to rationally resolve divergent opinions in critical discussions (Noroozi et al., 2012). The term “argument” in this paper refers to artifacts created by students to express and justify claims, whereas the term “argumentation” reflects the process of constructing these artifacts (Sampson and Clark, 2008).

Asterhan and Schwarz (2016) maintained that despite a growing body of research on learning through argumentation and domain-specific knowledge, research is undermined by inconsistent findings, primarily because of variation in the quality of the discourse. They put forward several reasons for the relatively scant experimental evidence for the effects of argumentation on domain-specific content learning. First, argumentation may not have an added value over other instructional activities when assessment focuses on factual knowledge and requires superficial knowledge rather than deep cognitive processing. Second, a prerequisite for studying the impact of argumentation on content learning is to elicit productive argumentative discussions, by using a carefully designed learning task that supports effective peer argumentation. Yet, Asterhan and Schwarz asserted that even well-planned activities may be only partly effective. To make the case for argumentation as a mechanism for learning, they suggested comparing it to other learning activities. One possibility is to use experimental research designs to compare the effects of argumentation-based instruction, to instructional activity that does not employ components of argumentation (Yeh and She, 2010), or dispute vs. deliberation (Felton et al., 2009).

An example of a didactical method that bridges argumentation, domain-specific knowledge, and moral values is the Values and Knowledge Education (VaKE) approach (Weyringer et al., 2022). VaKE is based on a number of theories identified with the constructivist approach to learning (Patry et al., 2013). These include: (1) Piaget’s (1976) cognitive scheme, Kohlberg’s (1984) moral development theory, and dilemma discussion (Lind, 2003); construction of moral reasoning schemes (Rest, 1979); and von Glasersfeld’s (1998) radical theory and (2) Vygotsky’s (1978) social-cultural constructivist theories. VaKE provides a teaching design that utilizes morality and values education with knowledge education, placing an emphasis on critical thinking in a problem-based learning environment. The students are presented with a dilemma. which entails teamwork and discussing the pros and cons of alternative solutions, while considering value aspects and finding information that is necessary to establish arguments. This approach exposes students to topics relevant to their profession, clarifies their value-related importance, and allows them to form an independent opinion while emphasizing practices of dialogue and argumentation. Despite the potential of VaKE for deepening students’ domain-specific knowledge, research thus far has chiefly been focused on the social, moral, or epistemic aspects associated with this approach (Keast and Marangio, 2015), students’ critical thinking regarding a moral dilemma (Pnevmatikos et al., 2019) and lifelong learning skills such as responsibility-taking (Pnevmatikos et al., 2016), or challenges associated with its implementation (Weyringer et al., 2012; Weinberger et al., 2016). Hence, whereas these studies show that argumentative skills can be honed through dialogic argumentation practices, they do not explore how this in turn may impact the learning of domain-specific knowledge.

### Domain-Specific Knowledge

Asterhan and Schwarz (2016) supported the claim that argumentation may improve domain-specific factual, conceptual, and procedural knowledge (Anderson et al., 2001). *Factual knowledge* refers to the basic elements related to the discipline which students must be acquainted with. It reflects a superficial understanding of content (Biggs, 1993; Biggs et al., 2001), however, it is considered the basis upon which the following types of knowledge are constructed (Anderson et al., 2001). *Conceptual knowledge* is more complex than factual knowledge and refers to the understanding of concepts, similarities, and patterns in factual knowledge components, and reflects a deep understanding of content (Biggs et al., 2001). Instead of rote learning, it is focused on how knowledge fits into larger perspectives (Blumberg, 2009; Wilson, 2016). *Procedural knowledge* pertains to knowing “how” to do something, for example how to employ methods of inquiry, or to use methods accompanied by criteria to achieve a specific learning goal (Anderson et al., 2001).

Weinberger and Fischer (2006) maintained that these types of knowledge can be achieved by encouraging students to build sequences of arguments that represent a knowledge-building cycle. Knowledge building in this process requires that learners construct arguments to justify their position. This facilitates self-explanation of the learning material. Counterarguments prompt learners to rethink their initial argument thus facilitating meta-cognitive activities. During the argumentation process, learners may acquire multiple perspectives on a problem, and be encouraged to apply the newly acquired knowledge to solve new problems.

### Concept Mapping and Argument Construction

Merely a few studies showed how computer-supported collaborative learning (Weinberger et al., 2017; Kimmerle et al., 2021) can be leveraged to support argumentative skills and knowledge construction. To address this lacuna, in the current study, a technology-enabled CM method was used to facilitate argumentation processes and investigate how this method may serve as a learning platform that supports students’ domain-specific factual, conceptual, and procedural knowledge and their academic efficacy.

A concept map is a visual organizer of knowledge that helps students enrich their understanding of a particular domain (Novak and Gowin, 1984). CM was found useful in enhancing 10th-grade students’ scientific inquiry course performance (Huang et al., 2022); improving ninth-grade students’ learning achievement (Li et al., 2021); affecting children’s learning achievement, critical thinking, and learning attitude (Liang et al., 2021); enhancing fifth graders’ (especially those with higher levels of critical thinking tendency) learning performance (Hwang et al., 2021a); and in improving eighth-grade students’ learning achievement, critical thinking tendency and problem-posing quality (Hwang et al., 2021b). A recently published systematic review on the roles, applications and trends of concept map-supported learning (Chang et al., 2022) showed the benefits of using concept maps to facilitate knowledge construction in collaborative learning and improve learning achievement and motivation.

In the context of higher education settings, Machado and Carvalho (2020) have reviewed the benefits and challenges of CM. According to their findings, CM promotes critical thinking (Conceicao et al., 2017); has a positive effect on exam scores (Aydogan and Ergun, 2016); allows the integration of theory with practice (Bressington et al., 2018); and enables meaningful learning (Greenberg and Wilner, 2015; Martin et al., 2015).

In the context of argumentation, CM is considered a valuable tool that can be utilized to scaffold argumentation through visualization—providing students with a coherent construction of an argument (Jonassen and Kim, 2010). For example, Si et al. (2019) explored the effects of argumentation with CM during medical problem-based learning on individual clinical reasoning. This ability was assessed through problem-solving performance and arguments that students constructed during individual clinical reasoning processes. Toulmin’s model of argument was utilized as a structure for arguments. The students constructed concept maps based on their argumentation about a case under discussion. The quality of arguments and clinical problem-solving performance were checked. The results provided evidence that utilizing argumentation with the concept map method during problem-based learning positively affected the development of clinical reasoning skills by students. Yet, as noted by these researchers and others (Alt and Naamati-Schneider, 2021), merely using CM is insufficient in eliciting students’ reasoning process and should be coupled with a sound pedagogical approach (e.g., problem-based learning) to elicit this learning outcome.

### Concept Mapping and Academic Efficacy

While CM has been shown to be an effective tool for facilitating learning, its benefit to students’ academic self-efficacy has been underexplored. Grounded in the self-regulation theory, which evolved out of Bandura’s (1977; 1986) social cognitive model of behavior, this concept refers to a personal belief in one’s ability to excel in an academic task or to accomplish a specific academic goal (Bandura, 1997; Eccles and Wigfield, 2002). Nonetheless, several researchers have gleaned some evidence that CM may contribute to students’ academic self-efficacy. For example, Chularut and DeBacker (2004) investigated the effectiveness of CM as a learning strategy with 79 students in English as a Second Language classrooms by using a pre-test–post-test research design. The findings revealed that the CM research group showed significantly greater self-efficacy and achievement gains from pre-test to post-test than did the control group. Similarly, Bressington et al. (2018) postulated that CM might promote meaningful learning and improve learning self-efficacy in Asian mental health nursing students. To assess their hypotheses, they utilized a quasi-experimental mixed-methods design. The study comprised CM (research) group and a conventional teaching methods (control) group. However, in contrast to their postulation, there were no significant differences in self-reported learning self-efficacy between the groups. The researchers explained the results by the small sample size utilized in their research.

### Computer-Supported Argumentation and Domain-Specific Knowledge

Merely a few studies showed how computer-supported collaborative learning (Asterhan and Schwarz, 2016) and Social Network Sites (SNS) like Facebook (Tsovaltzi et al., 2017), can be leveraged to support argumentative skills and domain knowledge acquisition (domain-specific factual knowledge). Yet, there is, as yet, scant literature addressing the three domain-specific knowledge facets. For example, Valero Haro et al. (2019a) examined the effects of an online learning environment on bachelor students’ argumentative essay writing and domain-specific knowledge acquisition in the field of biotechnology. The participants analyzed a case and wrote an argumentative essay considering the advantages and disadvantages of genetically modified organisms. The results showed that the combination of worked examples and peer feedback in the online learning environment improved the quality of argumentative essay writing and facilitate the acquisition of domain-specific knowledge. However, it should be noted that the latter was measured using a questionnaire comprised of multiple-choice questions and one open question, aimed at evaluating students’ factual knowledge (e.g., “What is a continuous animal cell line?”).

Other studies focused attention on teachers’ scaffolding of argumentation competence and domain-specific knowledge acquisition. For example, McNeill and Krajcik (2009) investigated how different curricular scaffolds (context-specific vs. generic), affected middle school chemistry students’ learning of science content and their ability to construct scientific arguments. The context-specific scaffolds provided hints regarding the content knowledge to incorporate into the argument. The generic scaffolds supported students in understanding the general structure of an argument. Findings showed that the context-specific curricular scaffolds were more effective in supporting students in writing scientific arguments to explain phenomena, but only when explicit domain-general support was provided, hence both types of guidance are important in this learning process. Similarly, Valero Haro et al. (2019a) conducted a systematic review of research in secondary and higher education on the effects of different argument scaffolding aided by computer-supported collaborative argumentation on domain-specific knowledge acquisition. The authors claimed that students should receive argumentation theory (learning of argumentation) before engaging in a computer-supported collaborative argumentation thereby being aware of how to successfully construct knowledge and be able to transfer and apply this knowledge to future problem cases in the same or similar contexts. However, this transfer of knowledge was not tested or surveyed in these studies.

In a more recent study (Valero Haro et al., 2022), the researchers investigated the links between the components of argumentation competence (knowledge, behavior, and attitude), and domain-specific knowledge acquisition, in an online learning setting. Findings showed a significant relationship between argumentation behavior and domain-specific knowledge acquisition. The authors suggested that the capacity of students to transfer argumentation behavior to similar tasks can be attributed to their domain-specific knowledge acquisition. It should be noted that the individual acquisition of domain-specific knowledge measurement lacked the three non-hierarchical types of domain-specific factual, conceptual, and procedural knowledge (Anderson et al., 2001).

The same lucuma appears in Ackermann and Kavadarli’s (2022) study addressing argumentation on societal problems in the economic domain. Their sample included school students’ written arguments on problems in two performance tests by applying a domain-specific analytical framework that combined domain-general aspects (i.e., quality criteria of argument structure) and domain-specific aspects (i.e., quality criteria of argument content). Yet, the latter did not refer to the three types of domain-specific knowledge, but rather to specific aspects of argument content such as appropriateness (level of topical relevance in reason), or reference (type of reference in reason).

In their meta-analysis, Wecker and Fischer (2014) explored the role of the quality of argumentation for domain-specific knowledge gains in computer-supported collaborative learning settings. The mean effect of the argumentation interventions on domain-specific knowledge appeared to be small to non-existent. As in the above-mentioned studies, this study was not centered on the three types of domain-specific knowledge (Anderson et al., 2001), but rather classified domain-specific knowledge as types of a knowledge test, for example, items with an open answering format (such as essay-type questions or problem-solving-tasks) or multiple-choice items. Similarly, other studies focused attention on students’ argumentative writing and their understanding of the components of argumentation (Yaman, 2018), their ability to understand complex arguments (von der Mühlen et al., 2019), or their conceptual understanding and the growth of their writing skills (Chen et al., 2020). To address this lacuna, in the current study, an argumentation process was enabled by digital CM and applied in a course. We investigated how this method may serve to support students’ domain-specific factual, conceptual, and procedural knowledge.

### This Study

Based on the literature review, the first purpose of our research was to design an activity that enables higher education students to construct an argument by using digital CM. Our second objective was to assess how the learning activity may support students’ domain-specific factual, conceptual, and procedural knowledge compared with a traditional teacher-centered activity. To this end, assignment work produced and submitted by students was evaluated by the instructor (see section: “Domain-Specific Knowledge Evaluation”). The third purpose was to shed some light on how this designated digital CM activity might contribute to students’ academic efficacy. Accordingly, the following research questions and hypotheses were formulated:

(*Q1*) How effective is an argumentation-for-learning activity using digital CM in promoting students’ domain-specific factual, conceptual, and procedural knowledge? To assess the effectiveness of the activity in promoting students’ domain-specific knowledge, their (research group) domain-specific factual, conceptual, and procedural knowledge gains were compared with those of a control group enrolled in the same course but using a traditional teacher-centered approach to teaching. It was expected that higher scores will be attained by the research group on the three domain-specific knowledge factors (*H1*).

(*Q2*) How argumentation-for-learning activity with digital CM relates to academic efficacy? It was expected that students’ positive perception of the activity will be positively linked to their academic efficacy (*H2*). An effort was made to identify the most effective factors in the activity that contribute to students’ academic efficacy.

Accordingly, this quasi-experimental study included several steps. First, the learning activity was designed and activated. Next, the research group students’ perception of the activity and their academic efficacy were measured, and finally, analyses and comparisons of the research and control students’ domain-specific factual, conceptual, and procedural knowledge were conducted at the end of the course.

Background variables (e.g., prior domain-specific knowledge and individual differences in the skills of argumentation, Socioeconomic status [SES], age, gender, and ethnicity) were addressed to examine and control their potential effect on the research constructs.

## Method

### Participants

Two identical courses were selected for this research: the first course (the research group) with a total of 96 participating students, and the second course (the control group) including a total of 131 students. Both the research and control groups included second-year Social Science undergraduate students from a major multicultural college comprising Arab and Jewish students located in the periphery of Israel.

The students comprising the research and control groups were enrolled in two identical compulsory 13-week courses given in the second semester by the same instructor and research assistant. Quantitative data were gathered from students who chose to complete the questionnaires. The pre-test comprised 59 (research group) and 63 (control group). However, the final assignment was checked and analyzed for the total sample.

With reference to additional characteristics, Table 1 details the research and control groups’ characteristics (pre-test(. Non-significant between-group differences were found concerning the variable of gender, age, or ethnicity. SES was assessed by the student’s father’s educational attainment (FEA) and mother’s educational attainment (MEA). The most frequent MEA and FEA category in both groups was 3 = *high-school education.* Non-significant between-group results were found in these variables. Prior to obtaining participants’ consent, it was explained that the questionnaires were anonymous and that it was acceptable should they choose to return a partially completed questionnaire. Finally, participants were assured that no specific identifying information would be processed. The study was preauthorized by the college’s Ethics Committee.

**TABLE 1:** Research and control groups’ characteristics for the pre-test.

	Research *N* = 59	Control *N* = 63
Mean age	23.16 (*SD* = 6.23)	21.62 (*SD* = 1.81)
Gender	19% male students	11% male students
Culture	90% Arab students	96% Arab students

### Activity Design

The activity included three stages:

*Stage 1.* Before the activity, the students in both research and control groups received an identical task to examine their previous knowledge of the topic and argumentation skills.
*Stage 2.* Research and control groups learned the course contents, which included models for dealing with conflicts, through lectures and by reading academic articles. In this stage the students were exposed to conflict resolution literature: the Thomas-Kilmann Conflict Mode Instrument (TKI), which includes five major styles of conflict resolution: competing, avoiding, accommodating, compromising, and collaborating (Kilmann and Thomas, 1977; Shell, 2001), Adam Grant’s Give and Take Approach model for cooperation (Grant, 2013), the Difficult Conversations Approach (Stone et al., 2010), and Marshall Rosenberg’s Non-violent Communication Model (Rosenberg, 2015). The students were taught the material through lectures and by reading academic articles.
*Stage 3.* The intervention activity was introduced to the research group only. A dilemma was presented to them dealing with a conflict between a couple. They were asked to choose and describe how four of the previously taught models can either resolve the conflict or exacerbate it. In addition, they were asked to provide evidence for each argument based on reliable resources and explain at least two similarities or differences between the arguments. A digital platform (Mindomo) was used for the presentation of the arguments. In line with previous studies (Katharina et al., 2018; Valero Haro et al., 2019a) the students learned how to construct an argument before engaging in a computer-supported collaborative argumentation platform. The control group received assignments that included reading academic material and addressing related questions.

Moreover, to facilitate the assessment of their maps, the students were provided with well-established criteria, as illustrated in Table 2. This assessment tool was adapted from Panadero et al. (2013) to address the current study’s goals.

**TABLE 2:** Rubric for assessing the concept map.

Criteria/Score	4	3	2	1
Arguments and supporting information	All four arguments and justifications with supporting items of information are included.	Two-Three arguments and justifications with supporting items of information are included.	One argument and justification with supporting items of information is included.	Arguments and justifications with supporting items of information are incomplete and/or incorrect.
Hierarchy	The organization is complete and correct. The supporting information corroborates the arguments.	The organization is correct but incomplete. Most of the supporting information corroborates the arguments.	The organization is correct but incomplete. Most of the supporting information does not corroborate the arguments.	The organization is incomplete and/or incorrect.
Relationships among arguments/supporting information	Relationships were specified and well-explained.	Relationships were partly specified but explained.	Relationships were partly specified but not explained.	Relationships were partly or not specified and poorly/not explained.
Simplicity and easiness of understanding	The design is simple and easy to understand.	Some relationships are difficult to understand.	There is an excessive number of connections.	Neither the relationships nor the hierarchy are understandable.
				

The students were assisted by a teacher’s aide while working on their assignment. Assistance included individual support that involved clarifying concepts that were unclear to them and providing detailed explanations of how to create a concept map using the Mindomo platform. The instructor dedicated special lessons to guide students in completing the assignment.

**FIGURE 1:**
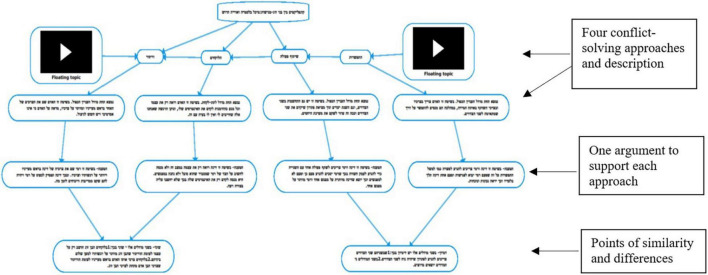
Arguments designed by using a *Mindomo* concept map.

As can be seen in Figure 1, the students chose four conflict-solving approaches and described each approach in detail. They then presented one argument to support each approach (represented by an arrow) and explained how the conflict could be solved by applying the approaches. The points of similarity and differences between two of the approaches are presented at the bottom of the figure. Here the students were required to elaborate on why the approaches are different or similar while providing a convincing explanation.

### Measurements

#### Domain-Specific Knowledge Evaluation

At the end of the semester, the students from the research group and the control group received identical assignments that revolved around a conflict situation that differed from the one given to the research group during the intervention program. This was done in order to assess the effectiveness of the intervention program in promoting domain-specific knowledge, in accordance with H1.

The conflict (Sabar et al., 2007) dealt with a pupil named Yosef who confided to his teacher about the difficult domestic and financial situation that he was dealing with at home. The teacher felt that the student was in need of consideration and support from the school system and weighed the possibility of sharing the information with other teachers to enable the school to support the student. On the other hand, the family’s problem and the student’s thoughts and feelings were confidential issues that were part of his private life, and informing other teachers about them would infringe upon his privacy.

The students received the following guidelines: The teacher and the student are involved in a conflict. How would you suggest that the teacher deal with the conflict? Choose two conflict resolution approaches that you have learned in the course as a basis for your suggestions.

1. Note the names of the two approaches you have selected and explain each of them according to what you have learned during the course (without any connection to the conflict).
2. Explain one difference and one similarity between the two approaches. Explain how the approaches are similar to each other and how they differ.
3. Explain how the teacher would be supposed to use each of the approaches that you chose. What will be the anticipated results for each usage?

The assignment was graded based on the identification of the use of three types of knowledge according to the taxonomy of Anderson et al. (2001): 1. factual, 2. conceptual, and 3. procedural. In this study, we compared the answers of students in the research group with those in the control group according to these three types of knowledge. Since these types of knowledge are complementary rather than hierarchical, each type of knowledge that was expressed in the student’s answer earned equal points. The examination was performed as an open-book test.

As described above, the assignment involved presenting two arguments for two approaches that the students selected. Accordingly, a complete answer with respect to each type of knowledge earned two points. One point (or half a point) was given for a partial answer, while an incorrect answer did not receive any points (=0). A complete answer, therefore, earned 6 points. Table 3 summarizes the grading method.

**TABLE 3:** Final assignment scores.

Score/Approach	Factual knowledge	Conceptual knowledge	Procedural knowledge	Total
The student fully referred to one approach (model)	1	1	1	3
The student fully referred to two approaches (models)	2	2	2	6

A complete answer with regard to factual knowledge involved a description of the approaches that were chosen to deal with the conflict. An example of this involved selecting two approaches from the Thomas–Kilmann Conflict Model of the competing approach (I win, you lose) and the collaborative approach (I win, you win). The following is a complete answer about the competitive approach which earned two points: “The competitive approach is expressed when we think of ourselves and rarely consider others. When we assume a competitive position, we attempt to provide only for our own needs without considering the ‘other.’ When each side defends only their position, it will be hard for them to find a solution. This is a classic conflict situation.” The following is a complete answer about the collaborating approach: “According to the collaborating approach, we take our needs, as well as those of others, into consideration. The approach is directed toward the self and the other on an equal basis, and both sides are balanced. There is an understanding that one cannot attain one’s own interests without considering the interests of the other as well.” Completely incorrect answers did not earn any points. Answers that earned partial points included correct but partial information about the approaches.

A complete answer regarding conceptual knowledge requires an understanding of the differences and similarities between the various approaches. For example, if students selected the competing approach of “I win, you lose” and the collaborating approach of “I win, you win” they had to emphasize the fundamental differences between the two approaches. The following is an example of a complete answer that earned two points: “These two approaches are significantly different from each other. One emphasizes struggle, competition, and a zero-end game with winners and losers, while the other emphasizes working together, cooperation, and attaining the objectives of both sides. People usually intuitively operate according to the competing approach, but if they work rationally, the collaborating approach will be better in many cases.”

Answers that were completely wrong did not earn any points. Answers that earned partial points were those that raised differences or similarities in a partial manner. An example of an answer that earned one out of two points dealt with a comparison between the collaborating approach and the avoiding approach: “The similarity between these two approaches is that the teacher is trying to help Yosef out of the goodness of her heart. The difference is that in the collaborating approach the conflict is solved by benefitting both the teacher and Yosef.” This answer involves a point of similarity that is not developed, while the point of difference relates to only one of the approaches.

A complete answer regarding procedural knowledge requires the application of two approaches that were chosen according to the information provided in the conflict. The following is an example of a complete answer that earned one point for the competing model:

In such a situation, the teacher must take action according to her own opinion without taking the student’s opinion into consideration. She will relate the information to the other teachers because there is no choice other than to do what her position requires her to do. In doing so, she will cause the other teachers to take Yosef’s situation into consideration. On the other hand, Yosef will be extremely angry and will remain dissatisfied.

An example of another complete answer described the collaborating approach:

The teacher will speak with Yosef, and they will decide together regarding which information will be included in the version to be shared with other teachers. No mention will be made of Yosef’s father or his mother’s illness. The only thing that will be mentioned is that Yosef is now responsible for many things at home and therefore she asks them to take his situation into consideration. In this way, both Yosef’s needs and those of the teacher will be expressed.

Answers that earned partial points were those that related to the application of only one approach or were unclear answers that were too short, answers in which the application was not compatible with the theoretical approach that the student chose, or answers in which the approach was only partially applied. The following partial answer that earned one point dealt with the application of the collaborating win-win approach:

The teacher has to take her needs and those of Yosef into consideration. This will attain her objective of helping Yosef and finding a solution that will promote both sides while preserving their relationship. Her objectives will be met, and she will be able to help Yosef.

In this case, the answer contains the beginnings of applying the approach, but there is no practical suggestion for dealing with the conflict. The collaboration approach requires action in which both sides will benefit, but the answer does not provide any suggestion for such action.

Answers that did not score any points were those that did not include any type of application, such as “the givers’ approach”:

Givers are wise people. People need to recognize their boundaries and to know what and how they can give in order that they can retain something to give themselves as well as to others later on. Despite all the limitations that people have, whether they are material or not, they can always find a way to give, contribute, and help without becoming empty.

This answer attempts to apply the “Givers” model but makes no reference to the case of the teacher and Yosef.

The following measurements were used to gauge the research-group students’ perceptions of the use of CM in the intervention and of their academic efficacy.

#### Concept Mapping for Problem-Based Learning Scale

This 12-item scale (Alt and Naamati-Schneider, 2021; Alt et al., 2022b) was designed to measure students’ perceptions of the effectiveness of using concept maps in the argumentation-for-learning process and to assess how it helped them during their decision making related to the dilemma, along with four factors: cognitive aspect, affective aspect, self-regulation of learning, and transfer of learning. The participants were asked to indicate their level of agreement with statements such as “Concept mapping helped me identify the interrelationships among arguments.” The items are scored on a six-point Likert scale ranging from 1 = *strongly disagree* to 6 = *strongly agree* (α = 0.97). A principal axis factoring analysis followed by a varimax rotation was used to corroborate the stability of the scale structure (eigenvalue > 1.00; item loadings > 0.30). The analysis solution accounted for 88.20% of the variance and yielded the above four categories. One item was omitted due to a low loading result on its ascribed factor (0.93 < α < 0.95).

#### Academic Efficacy

A five-item scale (Midgley et al., 2000) was used to assess perceived academic competence in the students’ learning environments. All items were scored on a 6-point Likert scale with anchors of 1 = *strongly disagree* to 6 = *strongly agree*. For example, “I’m certain I can master the skills being taught in this course” or “I can do even the hardest work in this class if I try” (α = 0.90).

Following the general guidelines for skewness and kurtosis (suggesting that if the number is greater than +1 or lower than −1, then the distribution is skewed, flat, or peaked, Hair et al., 2017), the distributions of Concept Mapping for Problem-based Learning Scale *(CM-PBL)* (skewness = −0.841, kurtosis = 0.025) can be considered normal. The distributions of *Academic Efficacy* (skewness = −0.943, kurtosis = 1.924), cannot be considered normal.

### Data Analysis

Students’ answers were analyzed by two expert raters in the research domain of conflict resolution and constructivist learning, the coding was performed blind to condition. An inter-rater Cohen’s Kappa (k) reliability method (Cohen, 1960), which is commonly used for assessment in psychological research, was performed. The raters were asked to categorize the students’ answers in the final assignment according to the theoretical scheme (Anderson et al., 2001). The k values were interpreted as follows: *k* < 0.20 poor agreement; 0.21 < *k* < 0.40 fair agreement; 0.41 < *k* < 0.60 moderate agreement; 0.61 < *k* < 0.80 good agreement; 0.81 < *k* < 1.00 very good agreement. Results of 0.61 < *k* < 1 were considered acceptable for the purposes of the current study.

Quantitative data were analyzed using a *t*-test, a multivariate analysis (MANOVA), and Partial Least Squares—Structural Equation Modeling (PLS-SEM; Hair et al., 2017). SmartPLS 3 software was used for this purpose. It should be noted that in situations where it is difficult to meet the strict requirements of more traditional multivariate techniques, such as normal data distribution, PLS-SEM is considered a preferred method. PLS-SEM has greater flexibility in this respect compared with covariance-based SEM (CB-SEM) when generally making no assumption about the data distribution. Therefore, data were analyzed by using PLS-SEM (Hair et al., 2017).

### Findings

#### First Research Question and Hypothesis

To evaluate the effectiveness of an argumentation-for-learning activity using digital CM in promoting students’ domain-specific factual, conceptual, and procedural knowledge, in accordance with *H1*, research and control group students’ answers in the final assignment were analyzed and compared in relation to the above three types of domain-specific knowledge. A complete answer on each level of knowledge earned two points; thus, it was possible to attain a maximum score of six points. The objective of the analysis of the answers was to assess the level of knowledge of the research group in comparison to the control group, according to *H1*.

**FIGURE 2:**
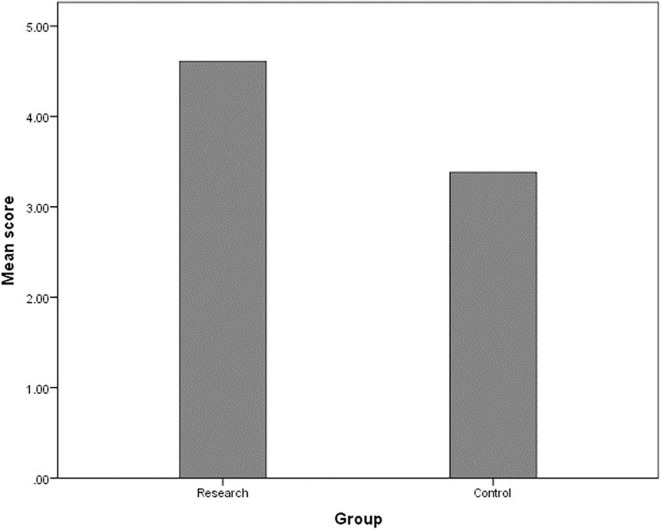
Between-group differences on three levels of knowledge (total mean score).

Figure 2 displays the overall scores in the comparison between the research group and the control group in the three types of knowledge. The average score in the research group was 4.6, while the average score in the control group was 3.4 (out of 6 possible points for a complete answer). The between-group differences regarding the overall scores were analyzed by using a *t*-test and were found statistically significant [*t*_(223)_ = 5.80, *p* < 0.001]. The lowest score in the research group was 1, while the highest was 6. The lowest score in the control group was 0, while the highest was 6. Thirty-nine percent of the research group (*N* = 96) received the entire 6 points in the summary assignment, while only 14% of the control group (*N* = 131) received the maximum number of points.

**FIGURE 3:**
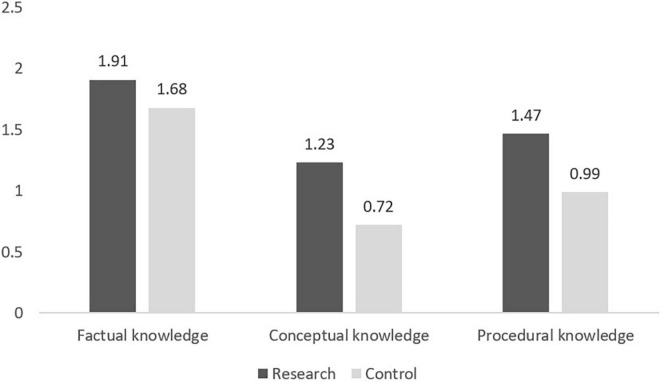
Between-group differences on three levels of knowledge.

A multivariate analysis (MANOVA) with Wilks’ Lambda criterion was applied to allow the characterization of differences between the groups (research vs. control) regarding a linear combination of the three dependent factors of knowledge. Table 4 displays the mean scores, standard deviations, *F*-values, and Eta-squared statistics of the two groups in the three factors. According to the results, the research group outperformed the control group in all three dependent factors [*F*_(3, 221)_ = 10.35, *p* < 0.001, η*_*p*_*^2^ = 0.123]. All the between-group differences were accompanied by low to moderate effect sizes (partial Eta-squared statistics η*_*p*_*^2^), whereby small, moderate, and large effects are reflected in values of η*_*p*_*^2^ equal to 0.0099, 0.0588, and 0.1379, respectively (Cohen, 1969, pp. 278–280; Richardson, 2011, p. 142). The highest partial effect size (η*_*p*_*^2^ = 0.092) was found for Procedural knowledge, and the lowest (η*_*p*_*^2^ = 0.045) for Factual knowledge. Figure 3 depicts the results. *H1* was corroborated.

**TABLE 4:** Mean scores, *SD*, *F*-values, and partial Eta-squared statistics (η*_*p*_*^2^) of the research and control groups.

Factors	Research group		Control group			
		
	*M*	*SD*	*M*	*SD*	*F*	η*_*p*_*^2^
Factual knowledge	1.91	0.29	1.68	0.62	10.60**	0.045
conceptual knowledge	1.23	0.82	0.72	0.85	20.21***	0.083
Procedural knowledge	1.47	0.69	0.99	0.81	22.68***	0.092

p < 0.01**, p < 0.001***.

#### Second Research Question and Hypothesis

**FIGURE 4:**
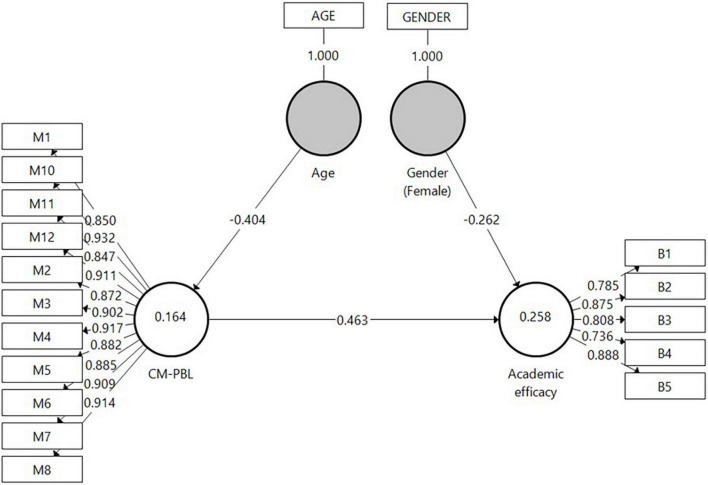
Model 1 analysis results of the examination of *H2* by SmartPLS.

In *H2* it was postulated that the research-group students’ perceptions of the intervention would increase their academic efficacy. To test this hypothesis, Model 1 (Figure 4) was designed. The model exemplifies two latent constructs: on the right, the Academic efficacy with its five indicators, and on the left, the perception of the CM for PBL factor, accompanied by its 11 indicators. Connections between the constructs as well as between the constructs and their assigned indicators are shown as arrows. A path was specified from the CM for PBL independent factor to the Academic efficacy dependent variable. It should be noted that based on a previously conducted analysis, background variables were also entered into the model to control their effect on the latent variables of age, gender, and ethnicity. Age and Gender were found significantly linked to the model’s constructs and, therefore, were included in the model.

A bootstrap routine was used to assess the direct effects as it makes no assumptions regarding the shape of the variables’ distribution or of the sampling distribution and can be applied to small sample sizes (Hair et al., 2017). As can be learned from Model 1, the CM for PBL factor was found positively connected to Academic efficacy (β = 0.463, *p* < 0.001). Regarding background variables, Age was found negatively connected to CM for PBL (β = −0.404, *p* < 0.001); i.e., older students were more reluctant to embrace CM for PBL. Female students had less Academic efficacy than male students (β = −0.262, *p* < 0.01).

##### Model Evaluation

The coefficient of determination (*R*^2^) value was examined, whereby *R*^2^ values of 0.75, 0.50, or 0.25 for endogenous latent variables can be described respectively as substantial, moderate, or weak (Hair et al., 2017). *R*^2^ for Academic efficacy was found weak (0.258). In addition to measuring the *R*^2^ values, the change in the *R*^2^ value when omitting a specified exogenous construct from the model was used to evaluate its impact on the endogenous constructs. This measure is referred to as the *f*^2^ effect size whereby values of 0.02, 0.15, and 0.35, respectively, represent small, medium, and large effects (Cohen, 1988). The *f*^2^ effect size result was 0.286 for CM for PBL and Academic efficacy. The predictive relevance (*Q*^2^) of the path model was evaluated by the blindfolding procedure (*Q^2^ v*alues should be larger than 0). The *Q*^2^ value of Academic efficacy in the present study was 0.138.

**FIGURE 5:**
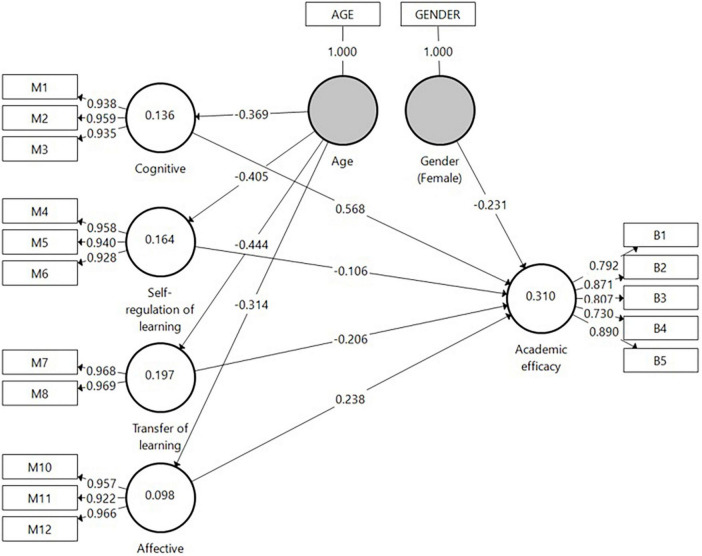
Model 2 analysis results of the examination of *H2* by SmartPLS.

To further examine *H2*, Model 2 (Figure 5) was designed. The model includes the same constructs as in Model 1; however, this time CM for PBL’s sub-factors (Cognitive, Affective, Self-regulation of learning, and Transfer of learning) were used to determine the effect of each of these sub-factors on the dependent variable of Academic efficacy. Paths were specified from CM for PBL independent sub-factors to Academic efficacy. Age and Gender were found significantly connected to the model’s constructs and, therefore, were included in the model. To test the direct effects, we ran the bootstrap routine. As can be learned from Model 2, only the Cognitive sub-factor of CM for PBL was found positively associated with Academic efficacy (β = 0.568, *p* < 0.01).

##### Model Evaluation

*R*^2^ for Academic efficacy was found to be moderate (0.310). In addition to measuring the *R*^2^ values, the change in the *R*^2^ value when omitting a specified exogenous construct from the model was used to evaluate its impact on the endogenous constructs. The *f*^2^ effect size result was 0.127 for Cognitive and Academic efficacy. Finally, the blindfolding procedure was used to assess the predictive relevance (*Q*^2^) of the path model. The *Q*^2^ value of Academic efficacy in the present study was 0.160.

In summary, the second hypothesis (*H2*) was confirmed insofar as the intervention was found effective in promoting students’ academic efficacy. However, the only significant impact was ascribed to the Cognitive sub-factor of the CM for PBL construct, whereas other sub-factors (Affective, Self-regulation of learning, and Transfer of learning) were found non-significant in predicting Academic efficacy.

## Discussion

Argumentation is deemed one of the most valued learning outcomes, particularly in higher education programs (Andreas et al., 2018), yet research on how this method may serve to support students’ domain-specific factual, conceptual, and procedural knowledge, and may contribute to students’ academic efficacy, is only in nascent stages. Therefore, our task was to evaluate the effectiveness of an argumentation-for-learning activity with digital CM in promoting students’ domain-specific factual, conceptual, and procedural knowledge, compared with a traditional teacher-centered activity. Another purpose was to evaluate the impact of the activity in promoting students’ academic efficacy.

Based on the findings, the research group outscored the control group enrolled in the traditional teacher-centered activity in all three domain-specific factual, conceptual, and procedural knowledge. While past studies (Schwarz and Baker, 2017) stipulated those argumentative skills can be advanced, to some extent, through constructivist-based activities, only scant research has been dedicated to empirically exploring how this in turn may impact students’ domain-specific factual, conceptual, and procedural knowledge (Valero Haro et al., 2019a,^2022^; Ackermann and Kavadarli, 2022). By comparing the effects of different task instructions, with and without an argumentation component (Yeh and She, 2010), the current research findings underline the superiority of the newly designed activity over the traditional teacher-centered approach in advancing students’ facets of domain-specific knowledge.

Another important finding is that the designed activity was less effective in reinforcing the factual knowledge domain pertaining to the essential elements students must know in order to be acquainted with a discipline or solve problems, and attain at least a surface understanding of content (Biggs et al., 2001). This can be corroborated by previous research findings (Asterhan and Schwarz, 2016) suggesting that argumentation may not have any particular added value in comparison to other instructional activities when the assessment process focuses on the surface understanding of content (e.g., factual knowledge), and does not require deeper cognitive processing. It is also plausible to infer that stating the names of the approaches and models of conflict resolution was a relatively easy assignment designed to elicit factual knowledge, specifically when the list of the models and approaches was made available to the research and control groups during the assignment.

The designed activity was found more effective in developing the conceptual knowledge domain, deemed to be more complex than factual knowledge, by reflecting a deep understanding of content and centering on the interrelationships among the basic elements within a larger structure that enables them to function together (Blumberg, 2009; Wilson, 2016). The highest impact of the argumentation-for-learning with CM activity was on students’ procedural knowledge—a discipline-specific set of skills that includes knowledge of the criteria used to determine when to use various procedures (Anderson et al., 2001). The present study adds to the corpus of knowledge by providing empirical evidence to the theoretical premise that argumentation processes may have a considerable effect on deep rather than surface knowledge aspects (Asterhan and Schwarz, 2016).

In addition, our findings demonstrated the benefits of the proposed designed activity in facilitating students’ academic efficacy. Indeed, previous studies (Chularut and DeBacker, 2004; Bressington et al., 2018) indicated the contribution of using CM to students’ academic self-efficacy. The current study, however, shows how this advantage can be harnessed in conjunction with an argumentation process to enhance students’ academic efficacy—an underexplored issue among studies that investigated the impact of computer-supported collaborative learning on argumentative skills. The attempt to reveal the most effective and contributive factor of the activity to students’ academic efficacy indicated that only the Cognitive sub-factor of the perceived CM in the argument construction process had a significant bearing on students’ academic efficacy. Hence, those students who reported that engaging in CM had helped them learn to identify the interrelationships between arguments, pinpoint these interrelationships, and learn more deeply about the topic felt more efficacious in mastering the skills being taught and more competent to succeed in the class work.

### Limitations and Future Research Directions

The perceived designed activity was found to be effective in enhancing students’ academic efficacy. However, the only significant impact was ascribed to the Cognitive sub-factor of CM in the argument construction variable, whereas other sub-factors (Self-regulation of learning, Transfer of learning, and Affective aspects) were found to be non-significant in predicting academic efficacy. Future studies should further hone and refine the model employed in this study by including additional motivational variables that might be linked to this model’s independent sub-factors, such as critical thinking (Conceicao et al., 2017), or self-regulation of learning (Naderifar, 2018).

Another limitation of this study is that it overlooked metacognitive knowledge (Flavell, 1992), pertaining to general strategies for learning and thinking. An attempt to foster metacognition was made by asking the students to write a reflective journal in Alt et al. (2022a) which they were instructed to narrate their self-perceived trajectory from their preliminary argument to a more complex one and to describe their challenges and gains in light of the experience. However, the improvement in metacognition over time was ultimately not evaluated. Examining students’ metacognitive knowledge with reference to the activity designed in this research might further explain how self-regulation can be improved through this activity.

## Conclusion and Implications

The results outlined herein stress the advantages of digital CM for designing arguments and underscore the advantages of this activity in advancing students’ domain-specific knowledge. It maintains that traditional and rote methods of learning are inefficient in eliciting students’ deep domain-specific knowledge; therefore, educators must find constructivist teaching methods to encourage students to make analytical thinking an intrinsic part of their daily practice. Educators need to use constructivist instructional strategies to equip students with knowledge in critical thinking, creative problem solving and collaboration (Chan, 2017).

In line with this notion, the proposed designed activity was proven to have contributed to the participant’s understanding of the structure of an argument. Moreover, it helped them recognize the importance of providing evidence and supporting facts to substantiate the reasoning offered to support their position and thus seems to have engaged them in a profound and meaningful learning process. This study thus establishes the assumption that combining constructivist teaching and learning methods with advanced technology, enables the development and acquisition of lifelong learning skills among students. Therefore, teachers should consider using authentic learning activities, involving real-world problems, such as VaKE, to enable students to explore, discuss, and construct concepts and relationships in contexts that are relevant to them. Such authentic problem-solving projects should aim at developing argumentative skills aided by collaborative e-platforms and encouraging high-order thinking levels. These learning processes include characteristics of critical thinking that enable students to formulate arguments, seek knowledge from reliable sources and evaluate information, search for alternatives, and collaboratively discuss alternative points of view. Teachers should be aware of the importance of integrating digital tools into constructivist teaching and harnessing the advantages of technology, and digital CM, in particular, to raise the quality of teaching and learning, and assessment in higher education.

Implications for conflict resolution studies can also be suggested. The students did not merely passively learn about conflict resolution theoretical models but also applied them to new situations through argument construction. A central goal of education is to provide learning experiences that are useful beyond the specific conditions of initial learning (Alt et al., 2020). Instructional methods that foster deeper initial learning successfully are those in which the students are asked to be more constructive in the initial learning processes. These methods typically foster deeper understanding, which leads to greater transfer, and enhances students’ knowledge beyond the information given (Lobato, 2003). As CM contributes to the integration of theory with practice (Bressington et al., 2018), the practice of conflict resolution might be applied by the students in different situations beyond those discussed in the classroom. Therefore, these activities may raise students’ awareness of more collaborative strategies of conflict resolution when faced with different types of conflict situations.

Nonetheless, the results ought to be interpreted with caution. A single instructional intervention might not yield adequate argumentation competencies, and, at its culmination, students may not yet be competent to transfer these skills to other problem-solving situations. These skills can be cultivated and honed through programs of recurrent practice in dialogic argumentation. When argumentation activities become an integral part of the classroom experience, they might also be transferred to other subjects (Kuhn and Crowell, 2011).

## Data Availability Statement

The datasets presented in this study can be found in online repositories. The names of the repository/repositories and accession number(s) can be found below: https://data.mendeley.com/datasets/9jyg9c3xt8/draft?a=040bf121-1cdd-4201-9167-7601f6c52aa9.

## Ethics Statement

The studies involving human participants were reviewed and approved by the Kinneret College on the Sea of Galilee. The patients/participants provided their written informed consent to participate in this study.

## Author Contributions

YK: conceptualization, data curation, writing–original draft preparation, and writing–reviewing and editing. DA: conceptualization, data curation, methodology, writing–original draft preparation, and writing–reviewing and editing. Both authors contributed to the article and approved the submitted version.

## Conflict of Interest

The authors declare that the research was conducted in the absence of any commercial or financial relationships that could be construed as a potential conflict of interest.

## Publisher’s Note

All claims expressed in this article are solely those of the authors and do not necessarily represent those of their affiliated organizations, or those of the publisher, the editors and the reviewers. Any product that may be evaluated in this article, or claim that may be made by its manufacturer, is not guaranteed or endorsed by the publisher.
